# Brief report: noise reduction in preschool from a self-regulated learning perspective—implementation of a game-based voice regulation training program

**DOI:** 10.3389/fpsyg.2023.1213348

**Published:** 2023-10-23

**Authors:** Lihi Sarfaty, Adar Ben-Eliyahu

**Affiliations:** Department of Counseling and Human Development, Faculty of Education, University of Haifa, Haifa, Israel

**Keywords:** psychological processes, self-regulated learning (SRL), preschool, behavior regulation, experiment, voice modulation, game-based intervention, language skills

## Abstract

An 8-week voice regulation training program (VRTP) incorporating everyday activities was implemented in an experimental preschool classroom (EG; *n* = 34), which was compared with a control preschool classroom (CG; *n* = 31). The VRTP includes songs, games, and conversations aiming to raise children's awareness of noise levels and teach voice modulation skills. Grounded in the theoretical framework of self-regulated learning, the study's objectives were to evaluate the impact of the VRTP on noise levels, children's self-regulation, and pre-literacy skills. Noise levels were assessed weekly using an electronic noise meter before and during the program. The EG preschoolers demonstrated modest but significant improvements over their pre-VRTP levels of voice modulation, behavioral and emotional self-regulated learning, and pre-literacy skills, in contrast with the CG children. The findings provide evidence that young children's self-regulation may be enhanced in preschool, challenging the field of developmental–educational psychology to consider self-regulated learning during early childhood.

## 1. Introduction

Vygotsky (1978) theory of learning presents a social and language-based approach to learning aimed at enhancing students' experiences as active speakers and listeners. Furthermore, Vygotsky emphasized that in order to internalize processes, young children express their self-talk out loud (Vygotsky, 1978). However, although children's learning relies on verbal communication, multiple children speaking simultaneously in the environment can cause an increase in speech volume in response to background noise (e.g., the Lombard effect; McKellin et al., 2011). Although we may expect a constant buzz in early-years classrooms when children are engaged in deep and meaningful learning, even through play, when this noise exceeds certain levels, it can hamper learning (Persson Waye and Karlberg, 2021). Within preschool settings, noise levels may exceed the recommended 50 dB (Heft, 2013), ranging from 58 to 72 dB (Shield and Dockrell, 2004), causing highly impaired hearing. Excessive noise levels interfere with auditory processing, memory, and attention, creating annoyance and motivational deficits (Maxwell and Evans, 2000). Very loud noise can disrupt children's learning, particularly given their limited linguistic resources, as the speech of conversational partners may become noise for unintended secondary audiences (McKellin et al., 2011; Hotchkin and Parks, 2013). Under a developmentally realistic perspective on the issue of noise in preschool classrooms, investment in decreasing noise levels should be considered, as these children (ages 3–6 years) are at a critical stage in acquiring the linguistic and social–emotional development required for formal schooling (Education Ministry, 2010; Office of Head Start, 2010). For example, children's vocabulary knowledge and phonological awareness may be mastered during the preschool years and serve as the foundation for reading comprehension in school and self-regulation (Anglin et al., 1993; Vallotton and Ayoub, 2011; Sala et al., 2014). *Vocabulary* refers to knowledge of words and their definitions. *Phonological awareness* is a term used to refer to the ability to identify and compare sounds; for example, to select a word that starts with a certain sound from among several words or to compare the sound with other sounds or different words (Education Ministry, 2007). Vocabulary and phonological awareness comprise pre-literacy skills.

Given that speaking is a behavior, high noise levels constitute a behavioral issue. Therefore, behavioral regulation—the ability to control and produce situationally appropriate actions and behaviors—is needed to reduce noise (Barbosa et al., 2022). Voice modulation interventions may be applied to control and modify inappropriate speech, speech frequency and duration, and voice intensity (Bronson, 2000; Fonagy and Target, 2002; Lee, 2005). Modifying noise levels becomes critical when the acoustic features of the classroom structure are ineffective in reducing noise (Christidou et al., 2015). Previous research investigating methods to reduce noise in the classroom have explored how the classroom's physical structure can affect noise levels. These interventions typically involve actions such as fitting sound absorbers, modifying floor carpets, and equipping chairs with noise-reducing covers (Evans, 2006; Persson Waye and Karlberg, 2021). For example, in their intervention study, Persson Waye and Karlberg (2021) changed the physical structure of seven preschools in Sweden (e.g., by changing floor mats to plastic mats). Using an electronic device that measures sound, they found slightly decreased sound levels in meal/craft- and playrooms. In investigating the voice regulation training program (VRTP), we were interested in identifying a noise reduction method targeting children's behavior rather than modifications to the classroom infrastructure.

The VRTP was designed to enhance psychological processes of the self-regulated learning (SRL) components of monitoring and controlling noise using age-appropriate game-like activities and circle games (Diamond et al., 2007; Barnett et al., 2008; Tominey and McClelland, 2011; Wijns et al., 2021). Given that SRL emerged from work on cognitive engagement in young adults (Corno and Mandinach, 1983; Winne and Hadwin, 1998; Panadero, 2017), applying SRL with young children from a developmental and educational perspective constitutes an innovation (Perry, 2019). SRL occurs in flexible and recursive stages as loosely sequenced cyclical feedback loops between monitoring and adjusting of emotions, behaviors, and cognitions as learners acquire knowledge or skills directed at achieving learning goals during studying or educational games (Zimmerman, 2000; Pintrich, 2004; Ben-Eliyahu, 2019; Compagnoni et al., 2019). *Cognitive SRL* (CSRL) refers to processes and strategies for monitoring and changing cognitions related to learning (e.g., information and memory processing). For example, a teacher who repeats letter names is applying and modeling rehearsal strategies. In preschool children, current work suggests that such learning strategies can be improved through interventions (Dörr and Perels, 2019; Wijns et al., 2021).

*Emotional SRL* (ESRL) refers to one's experiences, expression, and adjustment of emotions during learning (Ben-Eliyahu and Linnenbrink-Garcia, 2013). The most prominently studied forms of emotion regulation include *reappraisal* (thinking about the situation from another perspective) and *suppression* (not expressing emotion; Gross and John, 2003). These forms of ESRL have been found to shape emotions and the use of learning strategies (Ben-Eliyahu and Linnenbrink-Garcia, 2013, 2015). By age 5, children can recognize emotions, with marked improvements as they grow (Widen and Russell, 2013) and use autonomous strategies such as reappraisal; however, until age 5, preschoolers develop and regulate emotions with adult help (Sala et al., 2014).

*Behavioral SRL* (BSRL) refers to monitoring and changing of behaviors to achieve learning goals such as writing and talking. Regulation is maintained through self-management, environmental structuring, and knowledge about performing actions and behaviors (Zimmerman, 2000). BSRL is the first requirement when children enter school. They need to restrain or modulate many behaviors and their intensity, such as lowering their voices when working in groups or controlling impulsive behaviors (Lee, 2005; Savina, 2021). BSRL may manifest in several ways depending on age appropriateness: by planning where and what to learn; by initiating or stopping behavior (e.g., sitting still); by changing the intensity, frequency, and duration of actions; or by behaving appropriately in the absence of external monitoring (Thompson, 1991; Bronson, 2000; Zimmerman, 2000; Fonagy and Target, 2002; Berger et al., 2008).

Most SRL processes (e.g., working memory and attention) develop more or less in parallel, reaching maturity during adolescence (Pintrich and Zusho, 2002; Bryce et al., 2011). Despite their immature brain development, which leads to decreased abilities, preschoolers in most countries are expected to regulate their learning and engage in academic-type activities, such as identifying and naming colors, shapes, numbers, and letters, and dividing words into syllables (Education Ministry, 2010; McLean, 2010; Department for Education, 2017). To further confirm the validity of using the SRL framework to investigate preschool children's learning, we asked 45 preschool teachers to classify 47 activities their pupils engaged in as academic or non-academic (for a full description, see Supplementary material). The findings showed that academic learning occurs in preschool, ensuring the relevance of SRL for preschoolers.

As novice regulators, preschool children require scaffolding from others in order to regulate themselves successfully. Through teacher–student interactions, regulation in learning can be trained (Bronson, 2000; Diamond et al., 2007; Schmitt et al., 2015; Li et al., 2020). Thus, the preschool years are critical for developing regulation (Blair, 2002; Diamond et al., 2007; McClelland and Cameron, 2012; Barbareev, 2016).

Previous work has shown that regulation in specific domains can be trained, focusing on specific skills and examining their improvement (Diamond et al., 2007; Barnett et al., 2008; Tominey and McClelland, 2011). The innovation of the current study is that we investigated the efficacy of the VRTP (Research Question 1-RQ1) by comparing voice modulation in two groups: a preschool class that underwent the VRTP (experimental group [EG]) and a control preschool class [control group (CG)]. Furthermore, we sought to answer a basic scientific question (Research Question 2-RQ2): Does transfer occur from the behaviorally concrete operation of voice modulation to other SRL domains (e.g., CSRL) and pre-literacy skills? We hypothesized that EG participants would adjust their voices in different areas of the classroom more than the CG participants (RQ1). Second, we reasoned that if basic monitoring and control strategies are improved during the VRTP, then transfer might occur; thus, we hypothesized that improvements would occur in all SRL domains and pre-literacy skills (RQ2). Using an experimental design, we implemented a VRTP as an antidote for noise levels in early childhood formal education, merging developmental science and educational psychology by applying an SRL framework. Our primary goal was to discern how learning through play may lower noise levels in preschool and whether noise reduction can contribute to the development of children's self-regulation and language skills.

## 2. Methods

### 2.1. Participants

Two separate preschool classrooms serving children from a lower-middle socioeconomic demographic background in Israel, established in the past 5 years^1^, were recruited for the study and randomly assigned to one of the conditions. In Israel, educational institutions are neighborhood-based. In this way, preschools are allocated based on pupils' home addresses, resulting in a socioeconomically homogeneous group of families that can be characterized according to their community. The two preschool classrooms were chosen after consulting with the city's Department of Education to ensure their common features and a similar socioeconomic background. This decision was also supported by the socioeconomic classification system of the national Central Bureau of Statistics. This geography-based assignment facilitated the recruitment of preschools. The groups were comparable in age, with children of ages ranging from 2.9 to 3.5 years, *t*(63) = −0.58, *p* = 0.564. The EG preschool classroom (*n* = 34, mean age = 38.35 months, *SD* = 3.21, 47% girls) underwent the VRTP, whereas the other preschool classroom (CG; *n* = 31, mean age = 38.84 months, *SD* = 3.55, 48% girls) was unaware of the intervention and implemented their routine education program. Each preschool classroom was taught by a different teacher. Among the 4-person team of teaching staff in the EG, the head teacher had 15 years of teaching practice, while among the 3-person team of teaching staff in the CG, the head teacher had 22 years of experience. The staff members at both preschools were all women, ranging in age from 23 to 56 years (*n* = 7, mean age = 40.29, *SD* = 12.07), and had teaching experience ranging from 3 to 22 years (mean = 12.71, *SD* = 7). Parental consent and child assent were obtained, and the university's ethics committee approved the study, enabling all preschool children to participate.

### 2.2. Instruments

The study utilized a combination of collectively and individually administrated measures. Participants' SRL and pre-literacy skills were assessed individually, while noise intensity measurements were taken collectively (see Appendices A, B).

#### 2.2.1. Voice regulation training program

The VRTP included games, activities, and visual aids (see Appendix A) to enhance children's awareness of their personal and collective voices, as well as training and imparting voice regulation strategies (Christidou et al., 2015). After 3 h of training, the EG teacher implemented 11 weeks of VRTP sessions. First, the teacher discussed voice intensity with students using the visual aids of a voice meter and signs depicting noise levels considered appropriate for different areas in the classroom (Appendix A). For example, children were attuned to the relative quiet characterizing the reading area, whereas outside, children could talk loudly. Children were then introduced to the “volume button,” an imaginary button that controls one's voice volume. One can turn an imaginary “knob” to adjust one's voice to match different spaces and situations, subject to classroom conventions, as presented in pictures. The children were informed that they could request that another person (a teacher or child) adjust their volume button. Another form of training included games and songs, such as repeating rhythms in a whisper or aloud, depending on what the teacher signaled. The games were implemented 2–3 times weekly, while the songs were incorporated daily. Additionally, hand gestures were practiced during the activities as a signal to diminish or augment voice volume. Throughout the 11 weeks, the teacher provided constructive feedback and encouragement to motivate children to regulate their voices.

#### 2.2.2. Noise intensity measures

Sound level was measured in both classrooms using the same electronic noise meter (type: GM1356 Digital LCD Sound Level Meter 30–130 dB) with the standard scale of decibels, a logarithmic scale (Evans, 2006). Noise readings provided accurate minimum and maximum decibels within seconds, depending on the ambient noise level. We used an average noise level measurement reflecting the actual noise in the classroom, as maximum voice readings may be impacted by momentary extraneous noises, such as a falling object, and minimum voice readings may be impacted by the number of children in the space.

#### 2.2.3. SRL questionnaires

SRL measures included 19 items adapted from the Ben-Eliyahu and Linnenbrink-Garcia (2015) SRL scales. These items were modified in line with comments on the original items from three preschool teachers who were not involved in the study. This adapted questionnaire was used for teacher reports on each child's CSRL (Cronbach's α for T1, T2 = 0.94), ESRL (reappraisal: Cronbach's α for T1 = 0.91, T2 = 0.89; suppression: Cronbach's α for T1 = 0.73, T2 = 0.77), and BSRL (Cronbach's α for T1 = 0.96, T2 = 0.98). Items were presented on a 5-point Likert-type scale, ranging from 1 (*never*) to 5 (*always*) (see Appendix B). Teachers' reports were used because teachers spend extensive periods interacting with and observing their students and can provide reliable reports on their SRL (Hutchinson et al., 2021). Teachers can even identify shifts in the children's emotional states by observing behaviors such as deep breathing, self-talk, or changes in facial expression, body language, and vocal tone.

#### 2.2.4. Pre-literacy assessments

These included vocabulary and phonological awareness evaluations specifically created for preschoolers, with children's responses categorized as either demonstrating knowledge or not, with a maximum of 36 points awarded (Aram and Levin, 2002; Tavor, 2008). Each accurate answer was worth one point. The outcomes of both assessments were merged to create a single measure of pre-literacy skills for parsimony. The reason to aggregate pre-literacy skill data was to reduce the risk of type I errors and to provide a clear interpretation of the research findings.

### 2.3. Procedure

**Figure 1:**
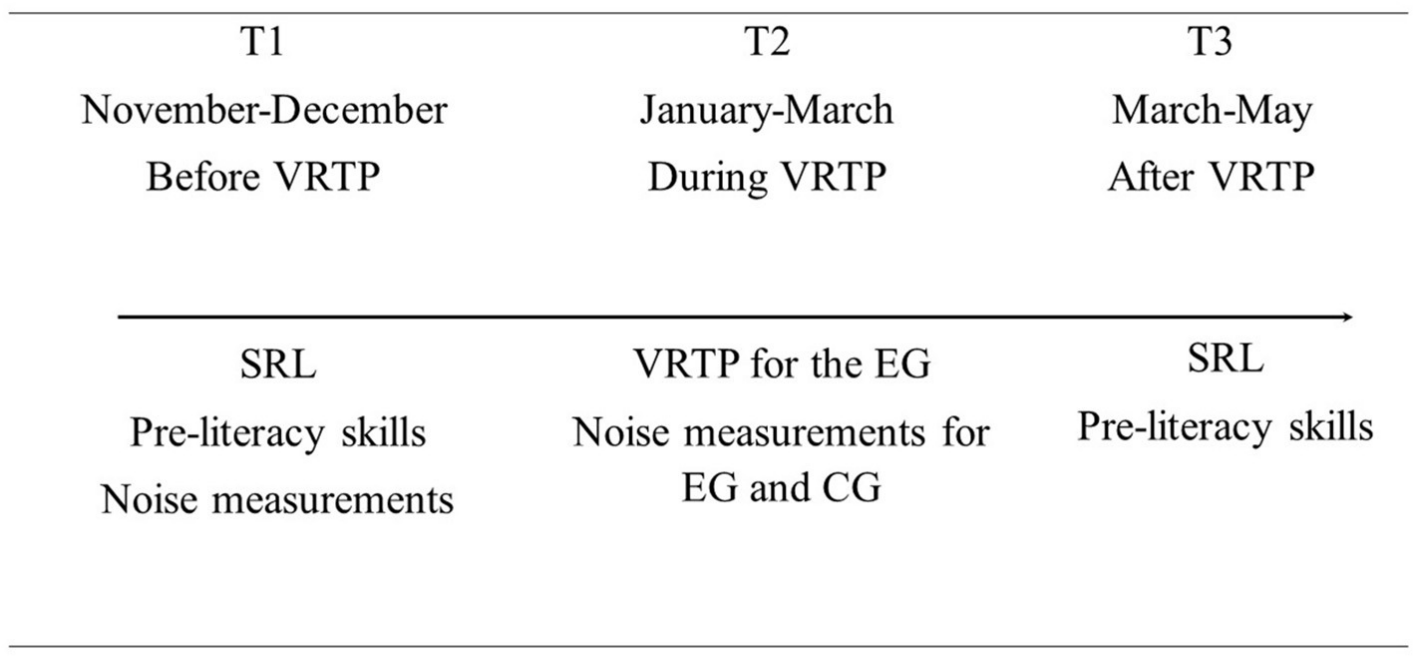
Timeline of study implementation and measurement design. EG, experimental group; CG, control group; SRL, self-regulated learning; VRTP, voice regulation training program.

The study was conducted during the school year. It consisted of three phases: 2 months at the beginning of the year for pre-training (T1), then 2 months of VRTP in the EG (T2), and a post-training evaluation at the end of training (T3). Figure 1 illustrates the study design and measurement timeline, showcasing the sequence of measures and intervals between evaluation points.

Noise intensity measures were carried out weekly in both preschool classrooms at T1 and T2. Measurements were taken in four classroom spaces in which varying noise levels were anticipated: the library, breakfast area, socio–dramatic play area, and all-purpose space (open space at the center of the classroom with tables for activities and didactic games). Data on noise intensity were collected seven times before implementation of the VRTP (T1) and eight times during its implementation (T2). To directly compare T1 and T2 measurements, we used mean substitution for the eighth T1 measurement (Tsikriktsis, 2005). To ensure standardization for both classrooms, noise intensity was recorded on Thursdays, when all staff members were present in both preschool classrooms. The sound level was recorded at the same time and in equivalent spaces (such as breakfast or free play spaces) in both classrooms (Keller-Bell and Short, 2019). Due to various school holidays during which fewer children were present, noise measures were not taken during T3, as some children were on extended trips with their families. These factors created fluctuations in the preschoolers' attendance and daily routines, making it difficult to obtain consistent and representative noise measurements at that particular time point. The unstable environment of the holidays and end–the-year atmosphere extended beyond the typical controllable factors and potentially would have brought bias into the results. The children's SRL measures and pre-literacy skills measures were collected at T1 and T3. Pre-literacy skill assessments were conducted by the first author in two 20-min sessions with each child separately.

Teachers were blind to the study design, questions, and to the regulation component. Teachers did not know how many other preschools were involved in the study or whether the training was part of the intervention. The teachers focused only on voice measurement.

### 2.4. Data analysis plan

The analyses addressed the effects of the VRTP on voice regulation (RQ1) and on SRL and pre-literacy achievement (RQ2). To evaluate noise levels, we compared the decibel levels in the EG and the CG at T2 using nonparametric tests for small sample sizes (Rosner and Glynn, 2009). Nonparametric tests were further justified as the assumption of homogeneity of variance was not met [Box's M: *F*(36) = 4.60, *p* < 0.001]. The Mann–Whitney (MW) *U*-test, an alternative to the two-sample *t*-test, was used to assess the differences (median-based) between and within groups for significance. Effect sizes were calculated using an online calculator that transforms the test statistic Z into effect sizes (Lenhard and Lenhard, 2016), and the G^*^Power program was used to estimate power. To examine interactions, we calculated a difference score for each group (e.g., T1 ESRL was subtracted from T3 ESRL), reflecting the degree of change in SRL and pre-literacy achievement across time and enabling investigation of the differences between the groups' respective progress. A positive score reflects an increase in capacity, whereas a negative score reflects a decrease.

## 3. Results

### 3.1. Voice intensity

MW tests were used to investigate differences in noise decibel levels between the EG and CG in all areas (based on a total score) and separately in each space (see Table 1). To establish baseline group differences and account for these, we examined noise decibel levels prior to the VRTP intervention (T1). At T1, the EG was characterized by lower noise levels (EG_median_ = 64.64, CG_median_ = 70.18). Examining the differences between the EG and CG for each space, EG noise levels were significantly lower than the CG noise levels in the library and the socio–dramatic play area. During implementation of the VRTP (T2), significant noise level differences were found in the all-purpose space, in addition to the differences in the library and socio–dramatic play area. The “all-purpose space,” the classroom's central area where children engage in play activities at set periods throughout the day, typically operates parallel to the library space but its use does not overlap with breakfast time. In the all-purpose space, the EG maintained lower noise levels over time and decreased noise levels during the VRTP.

**Table 1:** Differences in noise level (decibel) between experimental group (EG) and control group (CG) and within differences by activity area and for all areas together.

	**Between-groups tests** ^**a**^												**Test of within groups** ^**b**^							
	**Pre-training (T1)**		**Sig**.	**U**	***r***	**Power**	**During training (T2)**		**Sig**.	**U**	***r***	**Power**	**EG**				**CG**			
	**EG**	**CG**					**EG**	**CG**					**Sig**.	**Z**	***r***	**Power**	**Sig**.	**Z**	***r***	**Power**
Library			0.002	2.00	2.56	0.84			0.001	0.00	3.10	0.93	0.674	−0.42	0.21	0.70	0.208	−1.26	0.66	0.51
Median	62.19	72.32					61.05	70.65												
Range	14.70	10.10					26.80	8.25												
Breakfast			0.753	29.00	0.16	0.76			0.916	31.00	0.05	0.92	0.208	−1.26	0.66	0.51	1.00	0.00	–	–
Median	60.03	60.74					63.82	64.72												
Range	15.75	14.00					14.10	17.90												
Socio-dramatic play			0.018	9.50	1.46	0.57			0.002	2.00	2.56	0.84	0.123	−1.54	0.84	0.51	0.263	−1.12	0.58	0.51
Median	67.91	74.10					60.05	75.50												
Range	13.35	10.80					15.35	16.30												
All-purpose space			0.074	15.00	1.00	0.52			0.009	7.00	1.74	0.64	0.025	−2.24	1.35	0.56	0.161	−1.40	0.75	0.50
Median	69.55	74.00					68.07	75.42												
Range	19.75	10.80					15.20	16.30												
All areas together			0.006	6.00	1.87	0.65			0.001	0.00	3.10	0.93	0.069	−1.82	1.02	0.52	0.889	−0.14	0.07	0.89
Median	64.64	70.18					63.56	71.22												
Range	6.23	10.55					9.53	8.17												

^a^*p*-value for Mann-Whitney test. ^b^Test statistic (T) for Wilcoxon signed-ranks test. *r*, nonparametric Cohen's d based on an online calculator (Lenhard and Lenhard, 2016).

Examining each group for within-group differences at T1 vs. T2, the Wilcoxon signed-rank test yielded a significant difference between time points in the all-purpose space for the EG but not for the CG (Table 1). Considering minimum noise measurements, reflecting the lower-end potential for noise regulation and a baseline from which noise fluctuates in the classroom, a separate Wilcoxon signed-rank test exclusively on minimum intensities revealed no significant differences in the average noise level medians. Specifically, the EG showed a reduction in overall noise levels (Z = −1.960, *p* = 0.050. *r*_*effectsize*_ = 0.71) and a reduction in noise levels in the all-purpose space (Z = −2.100, *p* = 0.036. *r*_*effectsize*_ = 0.77). These findings suggest that the VRTP intervention was associated with decreased noise levels, at least in the noisiest areas of the preschool classroom.

### 3.2. Transfer effects: SRL and pre-literacy measure

Between-group and within-group differences were investigated (see Table 2). MW tests were conducted to compare the two groups on all measures prior to the VRTP intervention (T1), indicating that the EG had poorer BSRL and lower levels of reappraisal. After the VRTP (T3), the median suppression score in the CG was unchanged; however, significant differences indicated that the EG suppressed their emotions more than the CG after the VRTP but not before. Upon examining the overall SRL median scores, there was a significant difference between the groups before the VRTP (T1), with the EG having a lower median; however, after the VRTP (T3), no differences were observed between the groups, indicating that the overall SRL of the EG increased, closing the gap with the CG.

**Table 2:** Differences in SRL and academic achievement between the experimental group (EG) and control group (CG) and within differences for each group.

	**Between-groups tests** ^**a**^												**Test of within groups** ^**b**^							
	**Pre-training (T1)**		**Sig**.	**U**	***r***	**Power**	**Post-training (T3)**		**Sig**.	**U**	***r***	**Power**	**EG**				**CG**			
	**EG (*****n*** = **34)**	**CG (*****n*** = **31)**					**EG (*****n*** = **34)**	**CG (*****n*** = **31)**					**Sig**.	**Z**	***r***	**Power**	**Sig**.	**Z**	***r***	**Power**
BSRL			<0.001	95.50	2.00	1.00			<0.001	205.00	1.23	0.91	0.047	−2.12	0.55	0.55	0.152	−1.44	0.36	0.50
Median	1.00	3.00					1.00	2.40												
Range	0.60	4.00					4.00	4.00												
CSRL			0.358	457.50	0.23	0.52			0.301	448.50	0.26	0.51	0.061	−1.88	0.48	0.49	0.004	−2.79	0.74	0.47
Median	2.67	3.33					2.17	2.67												
Range	4.00	4.00					3.83	4.00												
ESRL; Reappraisal			<0.001	184.50	1.35	0.96			0.003	302.00	0.79	0.51	<0.001	−3.78	1.06	0.76	0.294	−1.07	0.27	0.52
Median	1.67	3.00					1.67	2.50												
Range	1.00	4.00					3.67	4.00												
ESRL; Suppression			0.298	457.00	0.23	0.47			<0.001	263.50	0.95	0.61	<0.001	−4.34	1.28	0.93	0.795	-.26	0.07	0.80
Median	1.00	1.00					3.00	1.00												
Range	3.67	4.00					4.00	4.00												
Overall SRL			<0.001	190.00	1.31	0.95			0.102	402.50	0.41	0.49	<0.001	−4.23	1.23	0.91	0.103	−1.63	0.41	0.49
Median	1.54	2.92					2.04	2.44												
Range	1.92	3.25					2.71	3.13												
Pre-literacy			0.249	439.50	0.29	0.50			0.457	470.50	0.19	0.56	<0.001	−4.64	1.41	0.98	0.012	−2.48	0.65	0.49
Median	16.50	18.00					19.50	19.00												
Range	21.00	19.00					22.00	19.00												

^a^*p*-value for Mann-Whitney U-test. ^b^Test statistic (T) for Wilcoxon signed-rank test. *r*, nonparametric Cohen's d based on an online calculator (Lenhard and Lenhard, 2016). BSRL, behavioral SRL; CSRL, cognitive SRL; ESRL, emotional SRL.

A sign test was used to examine within-group differences for each group, comparing T1 with T3. Among the EG children, changes in all parameters, aside from CSRL, which remained stable, were found to be highly significant (*p* < 0.001); in contrast, among the CG, only pre-literacy skills demonstrated growth, along with a decline in CSRL.

Considering differences before and after the intervention in each group (time × group interaction), we compared the difference scores for the raw data (T1 vs. T3) between the groups. In this way, for each measure, the gap between T1 and T3 was calculated (e.g., T1 ESRL was subtracted from T3 ESRL), meaning that a positive score indicated an increase over time and a negative score reflected a decrease. The raw score reflected the groups' respective progress, as measured by the change in SRL and pre-literacy skills across time. These difference scores were entered into the MW test. Significant differences were found between the groups (see Table 3) in BSRL, ESRL-reappraisal, ESRL-suppression, and pre-literacy achievement. These findings support the utility of the VRTP for enhancing BSRL, ESRL (reappraisal and suppression), and pre-literacy skills in the EG relative to the CG, providing support for the hypotheses.

**Table 3:** MW test results for between-groups T1–T3 difference scores.

	**EG**	**CG**	**Sig**.	**U**	***r***	**Power**
	**Median (range)**					
BSRL	0.00 (4.20)	0.00 (4.00)	0.005	341.50	0.63	0.35
CSRL	−0.25 (4.17)	−0.50 (4.17)	0.468	472.00	0.18	0.57
ESRL-reappraisal	0.42 (3.33)	0.00 (4.50)	0.008	324.50	0.70	0.51
ESRL-suppression	1.67 (4.33)	000 (5.67)	<0.001	233.00	1.09	0.79
Pre-literacy	4.00 (15.00)	1.00 (13.00)	0.001	285.50	0.86	0.48

^a^*p*-value for Mann-Whitney test. ^b^Test statistic (T) for Wilcoxon signed-ranks test. *r*, non-parametric Cohen's d based on an online calculator (Lenhard and Lenhard, 2016). BSRL, behavioral SRL; CSRL, cognitive SRL; ESRL, emotional SRL.

## 4. Discussion

The present study provides evidence that SRL could be integrated into the preschool curriculum. Thus, rather than older students' teachers having to undo maladaptive learning strategies or teach them how to learn, these strategies may be taught already in early childhood. Overall, the VRTP was found to be effective in reducing noise and in transferring to SRL and pre-literacy skills, providing partial support for the study's hypotheses. Voice regulation comprises habits linked to specific contexts, such as time, place, and the presence of people; to modify behavior, it is essential to focus on manipulating stable context cues rather than relying solely on willpower (Fiorella, 2020), facilitated by the VRTP. During preschool, as the entry point for children in learning how to behave in a school environment, learning social skills and voice regulation is crucial.

In addition to modest improvements in voice regulation, EG children demonstrated concurrent improvements in BSRL (planning) and ESRL after the intervention. An intervention of this nature may yield a form of transfer or strengthening of the SRL contingency (monitoring and controlling), so that once change was realized in relation to voice, the children appeared to apply this to other forms of BSRL (i.e., planning) and ESRL. As with BSRL, ESRL has a concrete outcome—the experience and expression of emotion—that preschoolers can feel and observe in themselves and others. EG preschoolers' teachers reported greater use of the reappraisal and suppression facets of emotion regulation after the intervention. These findings suggest that the EG preschoolers may have learned to be more flexible and choose between regulatory strategies to align with their desired emotions; this critical adaptation has been reported by most current research in adult samples (Sheppes, 2020). Our findings provide hope that the building blocks of SRL, namely monitoring and control, may be improved intentionally, as revealed in prior work with different age groups (Tominey and McClelland, 2011; Sezgin and Demiriz, 2019; Bernacki et al., 2020).

Although language achievements occur naturally over time, our findings demonstrate that the children participating in the VRTP showed more improvements in their pre-literacy achievements than the CG preschoolers (as reflected in the T1 and T3 difference scores). The EG's improved pre-literacy achievement coincides with their modest decrease in noise. The voice regulation internalization process may have facilitated general language development and communication (De Bruin and van Gog, 2012; Blair and Raver, 2015; Lonigan et al., 2017). During the VRTP, the preschoolers were exposed to games and structured interactions using accurate and nuanced language, voice intonation, and phonology, perhaps enhancing their attunement to their surroundings and social interactions.^2^ More work should be conducted to unpack and explore the source of the observed differences. In addition to the VRTP, other environmental and psychophysiological factors, such as parental involvement, language exposure, and neurological factors, may have been at play. In addition to considering such contextual and personal characteristics, future work should employ a more robust design with larger samples and longitudinal follow-up to obtain a deeper understanding of the dynamics associated with implementing the VRTP.

## 5. Conclusion and impact

Implementing a VRTP to reduce preschool noise levels coincided with ESRL, BSRL, and improvements in pre-literacy skills, suggesting benefits for developmental trajectories beyond reducing noise levels. Future studies should aim to obtain additional measurements documenting each child's voice and longitudinal data on the quality of their school transition. However, the current study provides evidence for a transfer of SRL across domains at young ages, an important contribution to developmental–educational psychology. As part of the recent surge of interest in preschool SRL (Erdmann and Hertel, 2019; Perry, 2019), the present study suggests that SRL development in young children may be supported with everyday activities easily incorporated into the current curriculum without necessitating additional funds. Educators and parents may incorporate voice modulation games or fun and simple exchanges to encourage children's awareness of their personal and collective voices.

## Data availability statement

The raw data supporting the conclusions of this article will be made available by the authors, without undue reservation.

## Ethics statement

The studies involving humans were approved by the University of Haifa—Ethics Committee. The studies were conducted in accordance with the local legislation and institutional requirements. Written informed consent for participation in this study was provided by the participants' legal guardians/next of kin.

## Author contributions

All authors listed have made a substantial, direct, and intellectual contribution to the work and approved it for publication.

## Footnotes

^1^Both preschool classrooms had similar acoustical features aimed at reducing noise levels such as blackboards on the walls, tile floors, and white acoustical tiles on the ceilings.

^2^For example, in the “guard dog” game, a child sits in the middle of the circle and needs to guard an object with their eyes closed. They focus on sounds and verbal exchanges in order to guard the object from their peers in the circle, who are the “thieves.” In doing so, children learn to be more aware of their environment in the guard dog role and also learn to identify their peers' intentions as part of the group trying to steal the object, as they need to communicate without making noise.
