# Supplementary material for: Brief report: noise reduction in preschool from a self-regulated learning perspective—implementation of a game-based voice regulation training program
Source: Front Psychol. 2023 Oct 23;14:1213348. doi: 10.3389/fpsyg.2023.1213348 (PMC10626535; doi:10.3389/fpsyg.2023.1213348)
Supplement: Supplementary file 1 [file Data_Sheet_1.docx]

**Appendix A**

**The VRTP activities and visual aids**

**Voice Regulation Training Program (VRTP)** **Intervention Protocol**

| **Activities Timeline** | | **Type of Activity** |
| --- | --- | --- |
| November | First week | - Activity to increase awareness and impart strategies- "Voice Meter" - "The Parachute" |
|  | Second week | - Intonation morning song - Intonation Recitations – "Quietly, Quietly, A Cat Sneaks Up" - Auditory memory game |
|  | Third week | - "Moving Instruments": drum - "The Guardian Dog" |
|  | Fourth week | - Rhythm and Sound- imitation - “In a Whisper in a Shout” |
| December | First week | - "The Volume Button" - Rhythm and Sound: Rhythms repeated by using the body |
|  | Second week | - Intonation Morning Song - "Hearing test" - "The Guardian Dog" |
|  | Third week | Holiday (blinded for review) break |
|  | Fourth week | - Intonation Recitations: "I Have a House" - Rhythm and Sound: Rhythms repeated by using the body - "Moving Instruments": bell |
| January | First week | - "Knock, Knock, who I am and what is my name" - "The Parachute" |
|  | Second week | - Rhythm and Sound: Rhythms repeated with musical instruments - In a whisper in a shout |
|  | Third week | - Intonation morning song - "Moving Instruments": Marax Rattle |

**Description of Activities**

***Activities to increase awareness and impart strategies:***

"Voice Meter": A class discussion considers noise levels appropriate for different areas in the classroom. Then the teacher presents the voice meter sign and different pitch level cards. The cards are hung as signs in the classroom according to the noise level discussed in the class discussion. One set of cards is left mobile for free use.

"The Volume Button": An imaginary "knob" to adjust the voice to match different spaces and situations, subject to classroom conventions. The teacher demonstrates how a volume knob works in an audio device then the children practice turning the knob in different pitch level and according to the classroom conventions.

After conducting the initial activity involving the sound meter and/or volume button, the teacher may exercise discretion and revisit the subject, further discuss and engage children's practice. For example, when she notices gaps in understanding or when children not adhering to the rules of the agreement outlined on the cards.

***Training activities:***

Intonation Morning Song: The teacher alters the pitch level of the voice when singing (i.e. sing loudly or quietly).

Teacher: Good morning to all children

Kids: Good morning

Teacher: Good morning to all the girls

Girls: Good morning

Everyone: Sun is already shining; It's sends rays; Tap on your knees; To ____ (the name of the teacher) here is a noise

Teacher: Good

Kids: Morning

Intonation Recitations: "Quietly, Quietly, A Cat Sneaks Up"

Three level activity. 1. The recitation is performed only by the teacher who matches the pitch level to the sentence while patting her knees ("quietly" = slow and quiet, "jumps" = fast and loud). The children remain seated and imitate the teacher's movements. 2. After several times the teacher asks a child to replace her and conduct the game. 3. For the third time, the teacher offers the children to move in space like the cat and like the rabbit - to walk quietly so no one can hear the steps and then to jump and sing loudly aligned with the song words.

"Quietly, Quietly, A cat sneaks up"

"And a rabbit with both his ears jumps and plays"

Intonation Recitations: "I Have a House"- The teacher repeats the recitation several times, starting with whispering the recitations and gradually increases her voice to a shout, if possible, pointing to the proper volume at the sound meter. Every time she recites, she moves her hand in a way that illustrate the size of the house and create a parallel between whisper and small and between loud and big.

"I have a house - like this, like this "- The hands simulate walls and a roof when saying "like this"

"Out of it grows a flower - like this, like this, like this "- The hands show a flower growing

Sound and quiet games: "Auditory Memory Game" - the teacher prepares 10 identical opaque bottles filled with different materials while the children are watching. Every two bottles have the same material (water, stones, rice, sand, cotton wool). After preparing the bottles each child in his turn will try to identify pairs of bottles by hearing only.

Sound and quiet games: "Moving Instruments": while the children are sitting, they pass a musical instrument to a friend sitting next to them without making a sound. With each activity the level of complexity increases by the type of instrument, the number of instruments passed and by passing the instrument to a friend how sits across the room.

Sound and quiet games: "The Guardian Dog" - While the children are sitting in a circle, one child sits in a middle of circle with their eyes covered and an object laid in front of them on the floor. The child must guard the object by tracing the noise source and pointing at it while the other children try to take the object quietly.

Sound and quiet games: “Rhythm and Sound”: The children imitate and/or repeat a rhythm made by the teacher or another child while changing the volume of the produced rhythm. Rhythms can be played with musical instruments or by using the body. For example, clapping hands or stomping to a certain beat.

Sound and quiet games: "Hearing Test" in the socio-dramatic area- The "doctor" examines children’s hearing while they sit on a chair with their back to the doctor. They need to identify in which side the doctor is making noise.

Sound and quiet games: "Knock, Knock, Who I am and what is my name?" - While the children are sitting in a circle, the teacher invites one child to put their head on her lap and another child to gently tap on the first child's back, saying the phrase "Knock, knock, who I am and what is my name?" and return quietly to their chair. The first child must guess who tapped on their back and recited the sentence. For this game all kids must keep quiet.

"In a Whisper, In a Shout" - While the children are sitting in a circle, the teacher invite a child to look for an object hidden while he waited outside of the classroom. The children give the child directions by saying the name of the object in different pitch levels. Loud is getting close to the object and quiet is getting far. For example, if a ball is hidden the children will shout " Ball.. ball... " as the child gets closer to the ball and whisper as the child moves away from it.

"The Parachute" - an activity with a parachute. Music is played and the children lift the parachute or lower it according to the volume of the music that is played on an audio device.

**Visual aids: Voice meter and pitch level cards**


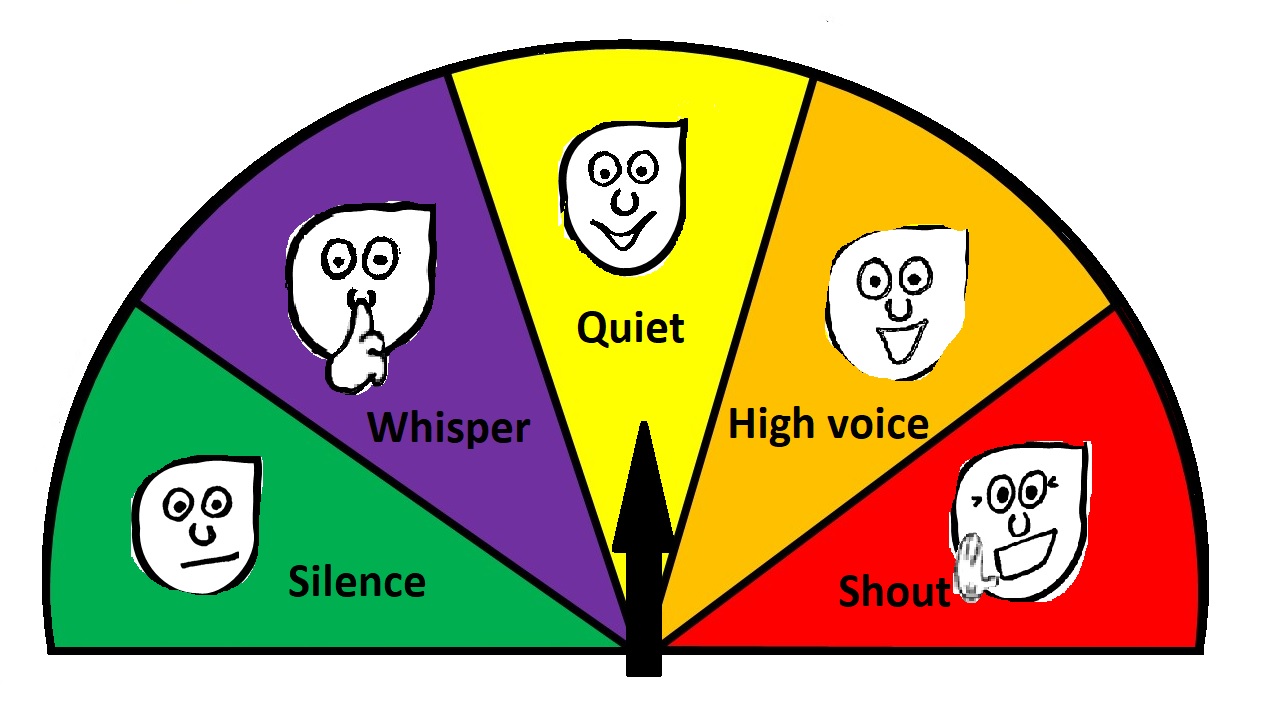

Whisper

Quiet

Silence


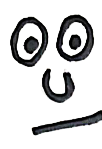

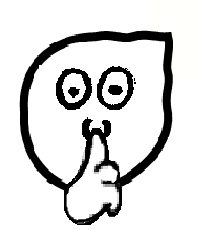


*High* *voice*

*Shout*


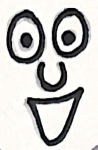

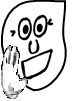


**Appendix B**

**Description of the Measures**

**Self-Regulated Learning (SRL) Questionnaire**

| ***Cognitive SRL (CSRL)*** |
| --- |
| 1. S/he has difficulty concentrating on tasks. |
| 1. S/he can't keep track of what s/he is thinking about during tasks. |
| 1. During learning, it is difficult for the child to think about one subject. |
| 1. S/he misses important information during learning because other things keep them busy (for example, additional tasks that need to be completed). |
| 1. S/he completes a task before moving on to another task |
| 1. S/he usually does a number of things at the same time, while s/he is working on academic assignments. |
| ***Emotional SRL (ESRL)*** |
| **Reappraisal** |
| 1. When s/he wants to feel less negative emotion (such as sadness or anger), s/he changes how s/he thinks; for example, s/he finds a different game or compromises. |
| 1. When s/he wants to feel a more positive emotion (such as joy or amusement), s/he changes how s/he thinks about the situation; for example, s/he changes it to something funny. |
| 1. When s/he wants to feel a more positive emotion (such as joy or amusement), s/he changes their thinking. For example, s/he finds a game that s/he likes. |
| 1. When s/he is facing a stressful situation, s/he makes themself think about it in a way that will calm them down; for example, s/he says calming words to themself, such as "it will be all right, I am a hero." |
| 1. S/he reduces negative emotion (e.g., sadness or anger) by changing the way s/he thinks about the situation. For example, explaining the situation with positive narrative. |
| 1. S/he controls their emotions by changing the way s/he thinks about the situation. |
| **Suppression** |
| 1. S/he controls their emotions by not expressing them. |
| 1. When s/he feels negative emotions, s/he makes sure not to express them (s/he does not express negative emotions). |
| 1. S/he keeps their emotions to themself. |
| ***Behavioral SRL (BSRL)*** |
| 1. S/he sets a plan for how to go about completing their assignments. For example, s/he says out loud what s/he will do. |
| 1. Before s/he begins a task, such as a drawing, s/he considers all the different things s/he needs in order to complete this task. |
| 1. S/he sets their daily agenda; for example, s/he determines what s/he will do in the afternoon (meet with a friend). |
| 1. S/he keeps track of what is left to do in his assignment. For example, s/he asks for more time to finish the task. |
| 1. S/he reminds themself or others of time. For example, s/he says to the teacher, "You said to take turns every five minutes. Five minutes had passed." |

**Pre-Literacy Achievement Assessment**

**Phonological Awareness Test (Aram & Levin, 2002)**

Identify the same bracket sound -

researcher: "Hello, we will play a game of words. I will say two words, you have to listen well, and tell me if the words end at the same sound."

"Let's try an example: " Sallahat "-" Orahat ". They both end with the same sound -'Hat'. "" Salla**hat** "-" Ora**hat** ""- but the word "Nahash" and the word "Arnav" doesn't. "Nahash" ends with 'hash' and "Arnav" ends with 'Nav'.

"Let's try another example: The words "Shaon" and "Maon". They both end with the same sound 'On'- but the word "Shemesh" and the word "Kadur" doesn't. "Shemesh" ends with 'Mesh' and "Kadur" ends with 'Dur'.

"Do the words Halon and balon end with the same sound?"

Repeat the question for all 10 pairs of words:

Hultza - Mafteah

Sabba - Abba

Shalom - Halom

Kisse - Sakin

Gerev - Kahol

Bait - Zait

Adom - Mazleg

Kohav - Layla

Mapa - Sapa

**Expressive Vocabulary Test (Tavor 2008)**

The child is asked to name the 26 pictures presenting nouns, verbs, adjectives, and adjectives. Due to copyright issue we cannot present the test itself.

**Supplemental Material**

The goal of this preliminary work was to establish that there is academic-type learning in preschool. Preschool teachers were asked to judge whether games were pre-academic or not.

**Design**

To help resolve the debate of whether academic learning transpires in preschool, a pilot study queried preschool teachers about the academic nature of common preschool activities. Provided that pre-academic learning occurs in preschool, we sought to investigate self-regulated learning intervention at young ages. Preschool teachers were asked to rate several preschool activities as reflecting academic or non-academic learning.

Method

**Participants**

Preschool teachers (*N* = 45; mean years of teaching = 11.20, SD = 7.06) completed a survey at their leisure. All the teachers were women (*M*_age_ = 38.5, SD =9.66). Most of the participants were head teachers at their preschool (80%). Nine of the teachers rotated among three fixed classrooms, working with a different age group every day. All teachers worked with children aged 3–6, with 14 teachers specializing in children aged 3–4. Ethical committee approval was received for this study, and teachers provided their written consent for participation. Data collection was anonymous.

Measures

Teachers were provided a list of 47 preschool activities (see Table 1) and were asked to classify each activity as either an academic or non-academic learning activity. We defined academic learning as "a set of activities aimed at developing school readiness skills, such as pre-literacy and pre-math, or intended to elicit skill-building and school engagement in the future, with the other activities being non-academic." Thus, the teachers rated each activity dichotomously––academic or non-academic.

Results

To determine whether activities should be categorized as academic, nonparametric binomial tests were run on teachers' ratings of their academic or non-academic categorization of each preschool activity, using SPSS version 26. This analysis determined the extent to which the ratings differed from chance. For example, games involving model building according to a given pattern were classified by most teachers as an academic learning activity (33 of 45; *p* =.003). In contrast, memory games yielded no significant difference, as 24 preschool teachers classified them as academic learning whereas 21 teachers classified them as non-academic learning (*p* =.766). All classifications and significance levels are presented in Table 1. This data substantiated that academic learning transpires in preschool, validating the use of SRL as a framework at this age.

Brief Discussion

Given some debate related to whether academic learning occurs in preschool, this pilot study enabled us to identify the activities that preschool teachers consider academic. Our findings indicated that most of the language and math games and games requiring focused attention were categorized as academic activities. In contrast, free play, social play, and motor activities, such as dramatic play and ball games, were considered non-academic activities. Whereas preschool may not be academic in the scholastic meaning, it deals with the nature of learning (Seng, 1998) as it comprises a classroom environment in which learning is required, such as acquiring basic concepts, arithmetic knowledge and the beginning of literacy (e.g., letter recognition) termed as *pre-academic skills* (Kernan and Hayes, 1998). These results highlight the critical value of investigating the development of SRL during the preschool years.

References

Kernan, M., & Hayes, N. (1998). Teacher expectations and the learning experiences of four‐year‐olds in preschool and primary school settings. *Irish Educational Studies*, 17(1), 222-240.‏

Seng, S. (1998, December 2-3). *Thinking about Thinking Skills in a Preschool Curriculum* [Conference presentation].‏ Singapore Conference on Preschool Education, Singapore. https://eric.ed.gov/?id=ED424961

Table 1

*Frequency of preschool teacher reports of preschool activities as academic or non-academic.*

| *p* value | Test statistic | Academic | Non-Academic |  |
| --- | --- | --- | --- | --- |
|  |  | *N* | *N* |  |
|  |  |  |  | **Academic activities** |
| .003 | 33 | 33 | 12 | 1. Model building according to a given pattern |
| .000 | 37 | 37 | 8 | 1. Path games that combine shape/ number identification and enumeration |
| .007 | 32 | 32 | 13 | 1. Hand-eye coordination games |
| .017 | 14 | 31 | 14 | 1. Sorting Games |
| .000 | 35 | 35 | 10 | 1. Experiments |
| .001 | 11 | 34 | 11 | 1. Listening to a story |
| .001 | 34 | 34 | 11 | 1. Exploration activity |
| .000 | 39 | 39 | 6 | 1. Meeting - Circle time |
| .003 | 33 | 33 | 12 | 1. Activity based on a story |
| .000 | 38 | 38 | 7 | 1. Morning meeting |
| .017 | 31 | 31 | 14 | 1. Day-end meeting |
| .003 | 33 | 33 | 12 | 1. Rhythm lesson |
| .000 | 38 | 38 | 7 | 1. Structured art based on a taught topic |
| .007 | 32 | 32 | 13 | 1. Group activity or plenary to promote emotional expression and coping in conflict situations |
| .003 | 12 | 33 | 12 | 1. Structured magnet game |
| .000 | 35 | 35 | 10 | 1. Physical education class |
| .000 | 38 | 38 | 7 | 1. Matching games- Quantity to quantity / Quantity to digit / Digit to digit |
| .000 | 38 | 38 | 7 | 1. Letter matching game |
| .000 | 39 | 39 | 6 | 1. Rhyme and syllable games |
|  |  |  |  | **Non-Academic Activities** |
| .003 | 33 | 12 | 33 | 1. Cube construction |
| .003 | 12 | 12 | 33 | 1. Threading games |
| .037 | 15 | 15 | 30 | 1. Peg board games, for example creative mosaic |
| .000 | 40 | 5 | 40 | 1. Magnets free play |
| .000 | 37 | 8 | 37 | 1. Household play |
| .000 | 35 | 10 | 35 | 1. Playing in the clinic |
| .000 | 40 | 5 | 40 | 1. Playing in the sandbox |
| .000 | 38 | 7 | 38 | 1. Model-free assembly games |
| .037 | 30 | 15 | 30 | 1. Social Game: Catch, "Simon said," etc. |
| .007 | 32 | 13 | 32 | 1. Playing in a sense box |
| .000 | 36 | 9 | 36 | 1. Unstructured creative art with various materials and colors |
| .000 | 40 | 5 | 40 | 1. Free play with dough |
| .000 | 36 | 9 | 36 | 1. Ball games |
|  |  |  |  | **Uncategorized Activities** |
| 1.00 | 23 | 23 | 22 | 1. Reading a book |
| .766 | 24 | 24 | 21 | 1. Memory games |
| .136 | 28 | 28 | 17 | 1. Lottery games |
| .136 | 28 | 28 | 17 | 1. Domino games |
| .371 | 26 | 26 | 19 | 1. Card games - Taki/War |
| .233 | 27 | 27 | 18 | 1. Cooking and baking |
| .766 | 21 | 24 | 21 | 1. Socio-dramatic play- selling and purchasing |
| .766 | 21 | 24 | 21 | 1. Vegetable garden care |
| 1.000 | 23 | 23 | 22 | 1. Group discussion, such as a 'tea house' |
| .371 | 19 | 19 | 26 | 1. Returning lunch box to bag |
| .551 | 25 | 20 | 25 | 1. Tiding the classroom and putting games back in place |
| .551 | 20 | 20 | 25 | 1. Motoric path games |
| .136 | 28 | 17 | 28 | 1. Building a puzzle |
| .766 | 21 | 21 | 24 | 1. Going to a show |
| .136 | 28 | 17 | 28 | 1. Cutting |
| *Notes*. Non-parametric binomial test summaries for teacher reports of categorization of preschool activities as academic or non-academic. | | | | |
